# Associations of the distance-saturation product and low-attenuation area percentage in pulmonary computed tomography with acute exacerbation in patients with chronic obstructive pulmonary disease

**DOI:** 10.3389/fmed.2022.1047420

**Published:** 2023-01-04

**Authors:** Kuan-Yuan Chen, Hsiao-Yun Kuo, Kang-Yun Lee, Po-Hao Feng, Sheng-Ming Wu, Hsiao-Chi Chuang, Tzu-Tao Chen, Wei-Lun Sun, Chien-Hua Tseng, Wen-Te Liu, Wun-Hao Cheng, Arnab Majumdar, Marc Stettler, Cheng-Yu Tsai, Shu-Chuan Ho

**Affiliations:** ^1^Division of Pulmonary Medicine, Department of Internal Medicine, Shuang Ho Hospital, Taipei Medical University, New Taipei City, Taiwan; ^2^Graduate Institute of Clinical Medicine, College of Medicine, Taipei Medical University, New Taipei City, Taiwan; ^3^School of Respiratory Therapy, College of Medicine, Taipei Medical University, New Taipei City, Taiwan; ^4^Division of Pulmonary Medicine, Department of Internal Medicine, School of Medicine, College of Medicine, Taipei Medical University, New Taipei City, Taiwan; ^5^Division of Critical Care Medicine, Department of Emergency and Critical Care Medicine, Shuang Ho Hospital, Taipei Medical University, New Taipei City, Taiwan; ^6^Sleep Center, Shuang Ho Hospital, Taipei Medical University, New Taipei City, Taiwan; ^7^Department of Medical Research, Shuang Ho Hospital, Taipei Medical University, New Taipei City, Taiwan; ^8^Research Center of Artificial Intelligence in Medicine, Taipei Medical University, New Taipei City, Taiwan; ^9^Department of Civil and Environmental Engineering, Imperial College London, London, United Kingdom

**Keywords:** distance-saturation product (DSP), chronic obstructive pulmonary disease (COPD), low-attenuation areas (LAA), modified medical research council (mMRC) scale, acute exacerbation of chronic obstructive pulmonary disease (AECOPD)

## Abstract

**Background:**

Chronic obstructive pulmonary disease (COPD) has high global health concerns, and previous research proposed various indicators to predict mortality, such as the distance-saturation product (DSP), derived from the 6-min walk test (6MWT), and the low-attenuation area percentage (LAA%) in pulmonary computed tomographic images. However, the feasibility of using these indicators to evaluate the stability of COPD still remains to be investigated. Associations of the DSP and LAA% with other COPD-related clinical parameters are also unknown. This study, thus, aimed to explore these associations.

**Methods:**

This retrospective study enrolled 111 patients with COPD from northern Taiwan. Individuals’ data we collected included results of a pulmonary function test (PFT), 6MWT, life quality survey [i.e., the modified Medical Research Council (mMRC) scale and COPD assessment test (CAT)], history of acute exacerbation of COPD (AECOPD), and LAA%. Next, the DSP was derived by the distance walked and the lowest oxygen saturation recorded during the 6MWT. In addition, the DSP and clinical phenotype grouping based on clinically significant outcomes by previous study approaches were employed for further investigation (i.e., DSP of 290 m%, LAA% of 20%, and AECOPD frequency of ≥1). Mean comparisons and linear and logistic regression models were utilized to explore associations among the assessed variables.

**Results:**

The low-DSP group (<290 m%) had significantly higher values for the mMRC, CAT, AECOPD frequency, and LAA% at different lung volume scales (total, right, and left), whereas it had lower values of the PFT and 6MWT parameters compared to the high-DSP group. Significant associations (with high odds ratios) were observed of the mMRC, CAT, AECOPD frequency, and PFT with low- and high-DSP groupings. Next, the risk of having AECOPD was associated with the mMRC, CAT, DSP, and LAA% (for the total, right, and left lungs).

**Conclusion:**

A lower value of the DSP was related to a greater worsening of symptoms, more-frequent exacerbations, poorer pulmonary function, and more-severe emphysema (higher LAA%). These readily determined parameters, including the DSP and LAA%, can serve as indicators for assessing the COPD clinical course and may can serve as a guide to corresponding treatments.

## Highlights

– In this study, we explored associations of the distance-saturation product (DSP) derived from the 6-min walk test (6MWT), pulmonary function test (PFT) parameters, emphysema severity (percentage of low-attenuation areas, LAA%), and dyspnea sensation, with the quality of life in patients with chronic obstructive pulmonary disease (COPD).
– We retrospectively collected a COPD dataset from northern Taiwan from July 2016 to April 2021.
– DSP was significantly associated with a reduced life quality and PFT parameters, elevated dyspnea sensation levels, and increased frequency of acute exacerbation of COPD.
– The low-DSP group (≤290 m%) had significantly higher values of the LAA% at various lung volume scales than did the high-DSP group (>290 m%).
– The DSP and LAA% can possibly serve as indicators to evaluate COPD stability.

## 1. Introduction

Chronic obstructive pulmonary disease (COPD) is a persistent illness that is characterized by repeated airway inflammation or obstruction, lung parenchyma (emphysema), and periodic deterioration of respiratory symptoms (1). World Health Organization (WHO) statistics in 2019 indicated that this disease was the third leading cause of death and the fifth leading cause of disability, and accounted for approximately 3.23 million deaths worldwide (2). COPD patients have a high risk of exhibiting dyspnea and experiencing excess mucus production because of emphysema or chronic bronchitis. Emphysema is associated with destruction of air sacs used for gas exchange in the alveoli, while bronchitis is related to inflammation of the airways (3). These clinical symptoms tangibly affect the quality of life (QOL) of COPD patients. In addition, since there are particular challenges for self-management by patients, acute exacerbation of COPD (AECOPD) is particularly likely to occur and is a common reason for hospitalization (4).

To assess the health status of COPD patients, the 6-min walk test (6MWT) is commonly employed in clinical practice (5). More specifically, the 6MWT, which contains the pulmonary function test (PFT) and exercise tolerance measurement, is able to provide cardiopulmonary parameters for patients with COPD. Previous studies investigated the association between parameters derived using the 6MWT and clinical manifestations of COPD. A relevant study, for example, indicated that the distance of the 6MWT presented a high ability to predict mortality caused by respiratory disease among patients with severe COPD (6). A cross-sectional study suggested that the distance of the 6MWT was associated with dyspnea sensations and QOL among patients with stable COPD (7). One study further analyzed oxygen saturation (SpO_2_) during the 6MWT and suggested that the model which considered both the distance and duration of SpO_2_ desaturation (defined as a fall in SpO_2_ of ≥4% or SpO_2_ of <90%) of the 6MWT had a higher ability to predict mortality compared to the model which only considered the 6MWT distance (8). Furthermore, other research employed a novel index, named the distance-saturation product (DSP), calculated by multiplying the distance of the 6MWT by the minimum oxygen saturation (SpO_2_), to predict mortality and suggested that the DSP was the strongest indicator of mortality (9). However, since the DSP is a novel index, associations of the DSP and parameters regarding the QOL with lung status parameters among COPD patients remain unclear.

Radiologically, high-resolution computed tomography (HRCT) is the main diagnostic tool for emphysema. Specifically, the low-attenuation area percentage (LAA%) derived from lung HRCT images is utilized to quantify emphysema severity (10). A previous study reported that this indicator was significantly and negatively associated with PFT parameters, including the forced expiratory volume in the first second (FEV_1_) and the FEV_1_/forced vital capacity (FVC) ratio (11). Prior research indicated that this indicator was significantly associated with the dynamic ventilatory response to exercise, such as the inspiratory capacity (IC) after exercise, among COPD patients (12). Another study revealed that this indicator was significantly correlated with responses to a QOL questionnaire and showed the ability to predict mortality at 3 and 12 months (13). Nevertheless, associations of the LAA%, AECOPD, and parameters of 6MWT with the novel DSP index still require further investigation.

This explorative study aimed to investigate associations among dyspnea sensation, QOL, parameters of the 6MWT, and the LAA% at various lung volume scales determined by HRCT among COPD patients. In addition, we examined the odds ratios (ORs) of the aforementioned measurements between the different grouped aspects, including (1) low- and high-DSP groups, (2) groups with and those without severe emphysema (LAA ≥ 20%), and (3) groups with and those without AECOPD. These were completed with the hope that the derived observations may provide further elucidation of relationships among these related lung function parameters and clinical presentations.

## 2. Materials and methods

### 2.1. Ethics

The Joint Institutional Review Board of Taipei Medical University (TMU-JIRB: N201902008) reviewed and approved the protocol of this retrospective study. All of the processes, including data collection, preservation, de-identification of recruited subjects, and further analyses, were conducted in accordance with the ratified procedure.

### 2.2. Study procedures and participant recruitment

This retrospective study collected a dataset from 850 patients diagnosed with COPD in the Pulmonary Medicine Department at Taipei Medical University’s Shuang Ho Hospital (New Taipei City, Taiwan) from July 2016 to April 2021. The recruitment criteria were (1) being aged 40∼85 years; (2) having been diagnosed with COPD per the Global Initiative for Chronic Obstructive Lung Disease (GOLD) criteria (14); (3) having undergone HRCT of the lungs; and (4) having completed the 6MWT and HRCT within a 6-month interval (with no particular examination order). It was noted that this COPD cohort excluded patients who exhibited other lung diseases (e.g., lung cancer, interstitial lung disease, and idiopathic lung fibrosis). There were 111 eligible subjects that were enrolled. Their demographic data, lifestyle status, and QOL questionnaire responses were obtained from medical records. Specifically, this study acquired their sex, age, body-mass index (BMI), tobacco use status (e.g., smoking status and calculation of pack-years), and the frequency of AECOPD during the prior year. Next, their responses were collected to various questionnaires, including the modified Medical Research Council (mMRC) scale and COPD assessment test (CAT). To assess the effect of measured outcomes from comorbidity, this study obtained their background of comorbidity and further calculated the Charlson comorbidity index (CCI) (15). It is worth noting that all of the questionnaire responses were collected from the interviews performed at the time of the last 6MWT (in a COPD stable stage). More precisely, participants were instructed to reply to all the questionnaires and then underwent the 6MWT. All of the derived data were used for further statistical examinations.

### 2.3. Measurement of pulmonary function

To determine the pulmonary function of recruited participants, this study collected both their PFT and 6MWT measurements. First, a spirometer (Vitalograph Spirotrac-5™, Vitalograph, MK18 1SW, Buckingham, UK) was utilized to measure the FEV_1_ and FVC, and then to derive the FEV_1_/FVC ratio. The measurement procedures were documented in previous studies (16, 17). Notably, all patients were instructed to stop using their bronchodilator, which was their regular medication, 1 day before the PFT. Next, during the examination, they were first provided with a bronchodilator (Short-acting beta-agonists-Fenoterol, 2-puff) and then underwent PFT. All of the PFT results of this study, thus, were collected after using a bronchodilator. The 6MWT was performed following clinical guidelines published by the American Thoracic Society (18). In more detail, patients were instructed to undergo a PFT to measure their baseline IC and then start walking at their own pace for 6 min. They were informed that the distance would be calculated as their score and were encouraged to walk as far as possible. The SpO_2_ during the 6MWT was measured *via* a wrist pulse oximeter, and a second IC measurement was performed immediately after the 6MWT was completed. The distance walked, oxygen saturation details, and IC data, namely measurements of the pre- and post-6MWT and their difference (ΔIC), were acquired. In addition, the DSP, calculated by multiplying the distance walked by the lowest SpO_2_ reading acquired during the 6MWT, was derived. All participants were separated into two groups according to the DSP of low DSP (DSP of ≤290 m%) and high DSP (DSP of >290 m%) for further investigation (9).

### 2.4. High-resolution computed tomography (CT; HRCT)

High-resolution computed tomography was conducted with a GE Discovery CT 750 HD (GE, Fort Myers, FL, USA), and each slice thickness was set to 10 mm. To acquire acceptable-quality images, all subjects were instructed to undergo one full inspiration and temporarily suspend their breath during the lung scan. To quantify emphysema severity, the LAA%, which is defined as less than or equal to the threshold (–950 Hounsfield units) on CT images, was determined by interpretation of certificated radiologists (19, 20). Emphysema severity was defined as normal (LAA: <5%), mild (LAA: 5∼10%), moderate (LAA: 10∼20%), and severe (LAA: ≥20%) (21). The current study obtained the LAA on various scales, including the total lungs, right lung, left lung, and individual lung lobes.

### 2.5. Statistical analysis

The present study employed SPSS statistical software to conduct all statistical examinations (SPSS vs. 20, Chicago, IL, USA). Descriptive statistics of the obtained variables were documented as the mean and standard deviation (SD) for continuous variables or number and percentage for categorical variables. As to statistical examinations, first, Student’s *t*-test was utilized to assess the mean differences in all derived continuous variables between the low- and high-DSP groups. The categorical variable of the tobacco use status was examined using a Chi-squared test. To investigate associations between the LAA at different lung volume scales (total, right, and left lungs) and characteristics of participants, including QOL questionnaire responses, PFT measurements, and 6MWT results, multivariable linear regression models with adjustments for age, sex, BMI, tobacco use status, and CCI were applied. The statistical results are reported as standardized beta coefficients with 95% confidence intervals (CIs). Next, this study utilized multivariable logistic regression models with and without adjustments for age, sex, BMI, tobacco use status, and CCI to investigate associations between the determined variables and the risk of being in the group with low-DSP, risk of developing severe emphysema (LAA ≥ 20%), and the risk of having AECOPD. More precisely, associations of responses for QOL, PFT parameters, and LAA values at various lung scales with the risk of being in the low-DSP group were investigated. Associations of responses for QOL and PFT and 6MWT parameters with the risk of severe emphysema were explored. Also, associations of responses for QOL, the PFT, 6MWT parameters, and LAA values at various lung scales with the risk of having AECOPD were surveyed. Results were documented as crude or adjusted ORs with 95% CIs. Statistical significance was set to *p* < 0.05.

## 3. Results

### 3.1. Demographics of recruited participants

Table 1 presents the demographics of enrolled patients who met the recruitment criteria. In total, there were 111 participants, including 37 in the low-DSP and 74 in the high-DSP groups. The mean age of the low-DSP group was significantly higher than that of the high-DSP group (*p* < 0.05). In terms of comorbidities, approximately 55% of the recruited patients had cardiovascular diseases (low-DSP: 54.05%; high-DSP: 56.76%) and about 19% had been diagnosed with metabolic syndrome. In more detail, nearly 20 and 14% of the recruited patients had a history of acute myocardial infarction and chronic heart failure, respectively. The mean value of CCI in the high DSP group (4.04 ± 1.20) was significantly lower than the values in the low DSP group (4.78 ± 1.69). Regarding the tobacco use status and COPD-related parameters, the low-DSP group demonstrated significantly higher means on the scores of the mMRC scale and CAT, and the frequency of AECOPD, compared to the high-DSP group (mMRC, CAT, and AECOPD all *p* < 0.01). Concerning the pulmonary function parameters of after using bronchodilator (Short-acting beta-agonists-Fenoterol, 2-puff), patients in the low-DSP group exhibited significantly lower means for PFT measurements, including the FEV_1_, FVC, and FEV_1_/FVC ratio, compared to measurements of patients in the high-DSP group (all *p* < 0.01). Similarly, for parameters obtained from the 6MWT, the low-DSP group had significantly lower walking distances and SpO_2_ values measured before and after the test compared to the high-DSP group (all *p* < 0.01). For IC alterations, the low-DSP group had a mean 0.13-L decrease in the ΔIC, whereas the high-DSP group had a mean 0.01-L decrease (*p* < 0.05). Regarding the LAA% determined by HRCT, patients with a low-DSP had significantly higher LAA mean values in the total lung, right lung, and left lung, compared to patients in the high-DSP group (all *p* < 0.05). In addition, only at the severe emphysema level (LAA ≥ 20%), low-DSP patients (*n* = 12) demonstrated higher values of the LAA compared with the 20 high-DSP patients (*n* = 20), and the mean difference was statistically significant (29.74 ± 6.68% vs. 24.45 ± 3.02%; *p* < 0.01).

**TABLE 1:** Demographic characteristics of subjects grouped by the distance-saturation product (DSP).

Categorical variables	All patients (*N* = 111)	Low DSP (*N* = 37)	High DSP (*N* = 74)	*p*
Age (years)^a^	69.14 ± 7.92	72.43 ± 7.56	67.49 ± 7.61	**0.0016**
Sex (male/female)^b^	99/12	31/6	68/6	0.1947
Body-mass index (kg/m^2^)^a^	23.36 ± 4.33	22.55 ± 4.71	23.76 ± 4.09	0.1660
**Comorbidities (*n*, %)^b^**				
Cardiovascular diseases	62 (55.86%)	20 (54.05%)	42 (56.76%)	0.7869
Acute myocardial infarction	22 (19.82%)	8 (21.62%)	14 (18.92%)	0.736
Chronic heart failure	15 (13.51%)	5 (13.51%)	10 (13.51%)	0.9999
Cerebrovascular accident	3 (2.7%)	0 (0%)	3 (4.05%)	0.535
Peripheral vascular disease	9 (8.11%)	2 (5.41%)	7 (9.46%)	0.144
Hypertension	21 (18.92%)	9 (24.32%)	12 (16.22%)	0.304
Depression and anxiety	6 (5.41%)	1 (2.71%)	5 (6.76%)	0.3732
Osteoporosis	2 (1.80%)	1 (2.71%)	1 (1.35%)	0.6138
Metabolic syndrome	21 (18.92%)	7 (18.92%)	14 (18.92%)	0.9999
CCI (score)	4.29 ± 1.42	4.78 ± 1.69	4.04 ± 1.20	**0.0299**
Tobacco use status (*n*, %)^b^				0.0667
Current smoker	53 (47.75%)	12 (32.43%)	41 (55.41%)	
Ex-smoker	50 (45.05%)	21 (56.76%)	29 (39.19%)	
Non-smoker	8 (7.20%)	4 (10.81%)	4 (5.41%)	
mMRC^a^	1.32 ± 1.04	2.19 ± 0.88	0.89 ± 0.82	**0.0001**
CAT^a^	9.26 ± 6.75	12.68 ± 7.95	7.55 ± 5.34	**0.0001**
AECOPD (times/year)^a^	0.66 ± 1.41	0.35 ± 0.92	0.34 ± 0.73	**0.0006**
**Pulmonary function test^a^**				
FEV_1_ (% predicted)	55.73 ± 20.15	42.19 ± 16.97	62.5 ± 18.18	**<0.0001**
FVC (% predicted)	77.90 ± 19.93	66.71 ± 18.39	83.49 ± 18.35	**<0.0001**
FEV_1/_FVC (%)	55.56 ± 10.68	48.58 ± 9.24	59.05 ± 9.63	**<0.0001**
**6-min walk test^a^**				
Distance walked (m)	374.01 ± 116.09	246.59 ± 84.6	437.72 ± 66.61	**<0.0001**
SpO_2–pre_ (%)	93.86 ± 2.43	92.51 ± 2.8	94.53 ± 1.92	**<0.0001**
SpO_2–post_ (%)	89.3 ± 5.44	85.41 ± 6.53	91.24 ± 3.48	**<0.0001**
ΔIC (L)	−0.05 ± 0.26	−0.13 ± 0.31	−0.01 ± 0.23	**0.022**
DSP (m %)	336.02 ± 110.77	210.07 ± 71.74	399.0 ± 62.38	**<0.0001**
**HRCT (%)^a^**				
Total lung LAA	15.30 ± 8.82	17.79 ± 10.17	14.06 ± 7.84	**0.0354**
Right lung LAA	15.03 ± 8.75	17.54 ± 10.13	13.78 ± 7.75	**0.0323**
Left lung LAA	15.63 ± 9.35	18.13 ± 10.47	14.38 ± 8.54	**0.0459**
**Emphysema severity^a^**				
LAA < 5% (*N* = 4, 10)	–	2.93 ± 1.52	2.63 ± 1.43	0.7388
5% ≤ LAA < 10% (*N* = 3, 18)	–	7.35 ± 1.88	8.17 ± 1.42	0.3840
10% ≤ LAA < 20% (*N* = 18, 26)	–	14.86 ± 2.87	14.54 ± 2.57	0.7075
LAA ≥ 20% (*N* = 12, 20)	–	29.74 ± 6.68	24.45 ± 3.02	**0.0044**

CCI, Charlson comorbidity index; mMRC, modified medical research council; CAT, chronic obstructive pulmonary disease assessment test; AE, acute exacerbation; FEV_1_, forced expiratory volume in the first second; FVC, forced vital capacity; HRCT, high-resolution computed tomography; LAA, low-attenuation area; SpO_2_, peripheral capillary oxygen saturation; IC, inspiratory capacity. Data are expressed as the mean ± standard deviation.

^a^Differences between groups were assessed by Student’s t-test.

^b^Differences between groups were assessed by a Chi-squared test.

*p*-values were measured by comparing between the low- and high-DSP groups.

Bold values mean that they had significant statistical meaning.

### 3.2. Associations of baseline values and PFT with emphysema severity between the low- and high-DSP groups

Table 2 presents the ORs of the responses for the QOL, PFT indices (measuring after using the bronchodilator), and LAA% at different lung volume scales between patients with low and high DSP. A 1-point increase in the mMRC scale and CAT scores was significantly associated with a 6.332-fold elevated OR (95% CI: 3.014∼13.303, *p* < 0.01) and 1.124-fold elevated OR (95% CI: 1.052∼1.201, *p* < 0.01) of presenting with low-DSP in the crude models. With additional adjustment for age, sex, BMI, tobacco use status, and CCI, a 6.162-fold elevated OR (95% CI: 2.737∼13.874, *p* < 0.01) in the mMRC scale and a 1.161-fold OR (95% CI: 1.07∼1.259, *p* < 0.01) in CAT scores of presenting with low-DSP were observed. A one-time increase in AECOPD was associated with a 2.05-fold elevated OR (95% CI: 1.277∼3.289, *p* < 0.01) of having low-DSP in the crude model and a 1.984-fold elevated OR (95% CI: 1.234∼3.188, *p* < 0.01) of having low-DSP in the model adjusted for age, sex, BMI, tobacco use status, and CCI. Regarding PFT parameters measured after employing the bronchodilator, increases of 1% in the FEV_1_ and FVC were significantly associated with decreased ORs of being in the low-DSP group (FEV_1_: crude model: OR: 0.937, 95% CI: 0.911∼0.964, *p* < 0.01; adjusted model: OR: 0.932, 95% CI: 0.902∼0.962, *p* < 0.01; FVC: crude model: OR: 0.95, 95% CI: 0.925∼0.975, *p* < 0.01; adjusted model: OR: 0.948, 95% CI: 0.921∼0.976, *p* < 0.01). For emphysema severity, a 1% increase in the LAA of the total and right lungs was significantly associated with a 1.05-fold elevated OR (95% CI: 1.002∼1.099, *p* < 0.05) and 1.051-fold elevated OR (95% CI: 1.003∼1.101, *p* < 0.05) of presenting with low-DSP in the crude model, but not in the adjusted model.

**TABLE 2:** Associations (odds ratios, ORs) of details of life quality, dyspnea sensation level, acute exacerbation frequency, pulmonary function test measurements, and low-attenuation area percentage (LAA%) between the low- and high-distance-saturation product (DSP) groups.

Categorical variables	Low DSP (*N* = 37) vs. high DSP (*N* = 74)	
	**Crude OR (95% CI)^a^**	**Adjusted OR (95% CI)^b^**
mMRC (point)	6.332 (3.014 to 13.303)**	6.162 (2.737 to 13.874)**
CAT (point)	1.124 (1.052 to 1.201)**	1.161 (1.07 to 1.259)**
AECOPD (time/year)	2.05 (1.277 to 3.289)**	1.984 (1.234 to 3.188)**
**Pulmonary function test**		
FEV_1_ (% predicted)	0.937 (0.911 to 0.964)**	0.932 (0.902 to 0.962)**
FVC (% predicted)	0.95 (0.925 to 0.975)**	0.948 (0.921 to 0.976)**
**LAA (%)**		
Total lung	1.05 (1.002 to 1.099)*	1.055 (0.997 to 1.116)
Right lung	1.051 (1.003 to 1.101)*	1.055 (0.997 to 1.117)
Left lung	1.044 (1.0 to 1.09)	1.048 (0.995 to 1.104)

CI, confidence interval; mMRC, modified medical research council; CAT, chronic obstructive pulmonary disease assessment test; AE, acute exacerbation; FEV_1_, forced expiratory volume in the first second; FVC, forced vital capacity.

^a^Simple logistic regression models.

^b^Multivariable logistic regression models adjusted for age, sex, body-mass index, tobacco use status, and Charlson comorbidity index.

*p < 0.05; **p < 0.01.

### 3.3. Associations of pulmonary function test with the LAA%

Table 3 summarizes outcomes of associations between the PFT (measured after using the bronchodilator) and LAA% at various lung volume scales. It was noted that all results were determined using a multivariable linear regression adjusted for age, sex, BMI, tobacco use status, and CCI. Increases of one point in the MRC and one event of AECOPD were significantly associated with increased LAAs in the total, right, and left lungs (all *p* < 0.01). As to measurements of the PFT and 6MWT, 1% increases in the FEV_1_ and SpO_2_ of the post-6MWT were significantly associated with LAA decreases in the total, right, and left lungs (all *p* < 0.01), while a 1% increase in the FVC was significantly associated with LAA decreases in the total and left lungs (*p* < 0.05). A 1-L increase in the ΔIC was significantly associated with declines in LAAs in the total, right, and left lungs (all *p* < 0.01). Similarly, each elevated unit (m%) of the DSP was significantly associated with decreases in LAAs in the total, right, and left lungs (all *p* < 0.05).

**TABLE 3:** Associations of details of the life quality, dyspnea sensation level, acute exacerbation frequency, pulmonary function test, and 6-min walk test with a low-attenuation area percentage (LAA%).

Categorical variables	Beta coefficient (95% confidence interval)		
	**Total lung LAA%**	**Right lung LAA%**	**Left lung LAA%**
mMRC (point)	2.296 (0.784 to 3.808)**	2.35 (0.878 to 3.821)**	2.207 (0.544 to 3.87)**
CAT (point)	1.465 (−0.025 to 2.955)	1.308 (−0.152 to 2.769)	1.637 (0.015 to 3.259)*
AECOPD (times/year)	2.044 (0.603 to 3.485)**	1.68 (0.257 to 3.104)*	2.815 (1.282 to 4.348)**
**Pulmonary function test**			
FEV_1_ (% predicted)	−3.325 (−4.768 to −1.882)**	−2.944 (−4.38 to −1.509)**	−3.799 (−5.355 to −2.243)**
FVC (% predicted)	−1.601 (−3.154 to −0.049)*	−1.335 (−2.86 to 0.19)	−1.97 (−3.651 to −0.288)*
**6-min walk test**			
Distance walked (m)	−1.485 (−3.156 to 0.186)	−1.488 (−3.119 to 0.144)	−1.395 (−3.222 to 0.432)
SpO_2–pre_ (%)	−0.275 (−1.809 to 1.259)	−0.169 (−1.669 to 1.331)	−0.556 (−2.225 to 1.113)
SpO_2–post_ (%)	−2.566 (−4.053 to −1.079)**	−2.437 (−3.894 to −0.979)**	−2.86 (−4.476 to −1.245)**
ΔIC (L)	−2.706 (−4.15 to −1.261)**	−2.445 (−3.87 to −1.02)**	−2.92 (−4.495 to −1.344)**
DSP (m%)	−1.922 (−3.597 to −0.247)*	−1.895 (−3.531 to −0.259)*	−1.891 (−3.724 to −0.058)*

mMRC, modified medical research council; CAT, chronic obstructive pulmonary disease assessment test; AE, acute exacerbation; FEV1, forced expiratory volume in the first second; FVC, forced vital capacity; SpO_2_, peripheral capillary oxygen saturation; IC, inspiratory capacity; DSP, distance-saturation product.

Multivariable linear regression models were adjusted for age, sex, body-mass index, tobacco use status, and Charlson comorbidity index.

*p < 0.05; **p < 0.01.

### 3.4. Associations of baseline values with the parameters of PFT and 6MWT between groups with and without severe emphysema

Table 4 lists the ORs of questionnaire outcomes, PFT (after using the bronchodilator) and 6MWT measurements between patients with and those without severe emphysema (LAA ≥ 20%: *n* = 32 vs. LAA < 20%: *n* = 79). An increase of 1% in the FEV_1_ was significantly associated with a 0.965-fold decline in the OR (95% CI: 0.943∼0.988, *p* < 0.01) of having severe emphysema in the crude model and a 0.965-fold decline in the OR (95% CI: 0.94∼0.99, *p* < 0.01) in the model adjusted for age, sex, BMI, tobacco use status, and CCI. Next, a 1-L increase in ΔIC was significantly associated with a 0.097-fold decline in the OR (95% CI: 0.018∼0.514, *p* < 0.01) of presenting with severe emphysema in the crude model and a 0.084-fold decline in the OR (95% CI: 0.012∼0.603, *p* < 0.01) in the model adjusted for age, sex, BMI, tobacco use status, and CCI.

**TABLE 4:** Associations (odds ratios, ORs) of details of life quality, dyspnea sensation level, acute exacerbation frequency, measurements of pulmonary function test, and the 6-min walk test between patients with and those without severe emphysema [low-attenuation area percentage (LAA%) ≥ 20%].

Categorical variables	LAA ≥ 20% (*N* = 32) vs. LAA < 20% (*N* = 79)	
	**Crude OR (95% CI)^a^**	**Adjusted OR (95% CI)^b^**
mMRC (point)	1.424 (0.953 to 2.127)	1.33 (0.833 to 2.123)
CAT (point)	1.062 (1.0 to 1.127)*	1.044 (0.976 to 1.116)
AECOPD (times/year)	1.151 (0.872 to 1.519)	1.082 (0.798 to 1.468)
**Pulmonary function test**		
FEV_1_ (% predicted)	0.965 (0.943 to 0.988)**	0.965 (0.94 to 0.99)**
FVC (% predicted)	0.989 (0.968 to 1.01)	0.986 (0.962 to 1.01)
**6-min walk test**		
Distance walked (m)	0.999 (0.995 to 1.003)	1.001 (0.997 to 1.006)
SpO_2–pre_ (%)	1.136 (0.943 to 1.368)	1.056 (0.867 to 1.286)
SpO_2–post_ (%)	0.933 (0.867 to 1.005)	0.899 (0.823 to 0.982)*
ΔIC (L)	0.097 (0.018 to 0.514)**	0.084 (0.012 to 0.603)*
DSP (m%)	0.998 (0.995 to 1.002)	1.0 (0.995 to 1.005)

CI, confidence interval; mMRC, modified medical research council; CAT, chronic obstructive pulmonary disease (COPD) assessment test; AE, acute exacerbation; FEV1, forced expiratory volume in the first second; FVC, forced vital capacity; SpO_2_, peripheral capillary oxygen saturation; IC, inspiratory capacity; DSP, distance-saturation product.

^a^Simple logistic regression models.

^b^Multivariable logistic regression models were adjusted for age, sex, body-mass index, tobacco use status, and Charlson comorbidity index.

*p < 0.05; **p < 0.01.

### 3.5. Associations of baseline characteristics with the PFT and 6MWT parameters, and the LAA% between groups with and without AECOPD

Table 5 reports the ORs of questionnaire outcomes, the PFT (after using the bronchodilator) and 6MWT, and the LAA% in the lungs (total, right, and left lung regions) between COPD patients with and those without AE (with AE: *n* = 38 vs. without AE: *n* = 73). First, as to questionnaire outcomes, one-point increases in the mMRC scale and CAT scores was significantly associated with increased ORs of experiencing AECOPD. Concerning PFT measurements, 1% increases in the FEV_1_ and FVC were significantly associated with reduced ORs of presenting with AECOPD (FEV_1_: crude model: OR: 0.956, 95% CI: 0.934∼0.98, *p* < 0.01; adjusted model: OR: 0.953, 95% CI: 0.929∼0.979, *p* < 0.01; FVC: crude model: OR: 0.964, 95% CI: 0.942∼0.987, *p* < 0.01; adjusted model: OR: 0.96, 95% CI: 0.935∼0.984, *p* < 0.01). Similarly, for 6MWT parameters, one-unit increases in the distance walked and DSP were significantly associated with decreased ORs of having AECOPD in both the crude and adjusted models (distance walked crude model: OR: 0.993, 95% CI: 0.989∼0.997, *p* < 0.01; adjusted model: OR: 0.992, 95% CI: 0.988∼0.997, *p* < 0.01; DSP crude model: OR: 0.964, 95% CI: 0.942∼0.987, *p* < 0.01; adjusted model: OR: 0.992, 95% CI: 0.987∼0.997, *p* < 0.01). Regarding emphysema severity, LAA values in the lungs (total, right, and left lung regions) were significantly associated with decreased ORs of presenting with AECOPD in both the crude and adjusted models.

**TABLE 5:** Associations (odds ratios, ORs) of details of life quality, dyspnea sensation level, pulmonary function test parameters, low-attenuation area percentage (LAA%), and 6-min walk test outcomes between participants with and those without having acute exacerbation (AE) of chronic obstructive pulmonary disease (COPD).

Categorical variables	With AE (*N* = 38) vs. without AE (*N* = 73)	
	**Crude OR (95% CI)^a^**	**Adjusted OR (95% CI)^b^**
mMRC (point)	2.506 (1.566 to 4.011)**	2.589 (1.56 to 4.297)**
CAT (point)	1.165 (1.082 to 1.254)**	1.173 (1.083 to 1.269)**
**Pulmonary function test**		
FEV_1_ (% predicted)	0.956 (0.934 to 0.98)**	0.953 (0.929 to 0.979)**
FVC (% predicted)	0.964 (0.942 to 0.987)**	0.96 (0.935 to 0.984)**
**6-min walk test**		
Distance walked (m)	0.993 (0.989 to 0.997)**	0.992 (0.988 to 0.997)**
SpO_2–pre_ (%)	0.907 (0.772 to 1.066)	0.888 (0.748 to 1.054)
SpO_2–post_ (%)	0.944 (0.879 to 1.014)	0.943 (0.873 to 1.018)
Δ IC (L)	1.993 (0.422 to 9.407)	2.746 (0.53 to 14.234)
DSP (m%)	0.993 (0.989 to 0.997)**	0.992 (0.987 to 0.997)**
**HRCT (%)**		
Total lung LAA	1.083 (1.031 to 1.138)**	1.083 (1.023 to 1.146)**
Right lung LAA	1.078 (1.026 to 1.132)**	1.077 (1.018 to 1.14)*
Left lung LAA	1.08 (1.03 to 1.132)**	1.078 (1.023 to 1.136)**

CI, confidence interval; mMRC, modified medical research council; CAT, COPD assessment test; FEV1, forced expiratory volume in the first second; FVC, forced vital capacity; SpO_2_, peripheral capillary oxygen saturation; IC, inspiratory capacity; DSP, distance-saturation product; HRCT, high-resolution computed tomography; LAA, low-attenuation area.

^a^Simple logistic regression models.

^b^Multivariable logistic regression models were adjusted for age, sex, body-mass index, tobacco use status, and Charlson comorbidity index.

*p < 0.05; **p < 0.01.

### 3.6. Supplementary analysis–associations between LAA% of each lobe, baseline values, PFT, and 6MWT

This study employed multivariable linear regression models adjusted for age, sex, BMI, tobacco use status, and CCI to explore associations among questionnaire outcomes, pulmonary function measurements, and LAAs at various lung lobe scales (Supplementary Table 1).

### 3.7. Supplementary analysis–independent risk factors for low DSP

This study employed two multivariable logistic regression models to explore independent risk factors for exhibiting DSP (Model-1: adjusted for age, sex, BMI, tobacco use status, CCI, lung function, symptoms, and LAA%; Model-2: adjusted for age, sex, BMI, tobacco use status, comorbidities, lung function, symptoms, and LAA%). Associations are summarized in Supplementary Table 2.

In the model-1, a 4.712-fold elevated OR (95% CI: 1.907∼11.646, *p* < 0.01) in the mMRC scale and a 0.934-fold declined OR (95% CI: 0.892∼0.977, *p* < 0.01) in the FEV_1_ of presenting with low-DSP were observed. Next, in the mode-2, a 1-score increased in the mMRC and a 1-L increase in the FEV_1_ was significantly associated with a 13.548-fold increase in the OR (95% CI: 3.289∼55.803, *p* < 0.01) and 0.913-fold decrease in the OR (95% CI: 0.859∼0.969, *p* < 0.01) of presenting with low DSP.

### 3.8. Supplementary analysis–associations among the DSP, baseline characteristics, PFT parameters, and LAA%

Summaries of the associations among the DSP, LAA%, baseline characteristics, and PFT outcomes (measured after using the bronchodilator) are presented in Supplementary Tables 3, 4, which were examined employing two types of multivariable linear regression models (Model-1: adjusted for age, sex, BMI, tobacco use status, and CCI; Model-2: adjusted for age, sex, BMI, tobacco use status, and comorbidities). In model-1, an increase in 1 unit of the DSP was significantly and negatively associated with the mMRC (−0.738, 95% CI: −0.902∼−0.573, *p* < 0.01), CAT (−3.633, 95% CI: −4.993∼−2.335, *p* < 0.01), and AECOPD (−0.694 times/year, 95% CI: −0.977∼−0.411, *p* < 0.01). In contrast, an increase of 1 unit in the DSP was significantly and positively associated with the FEV_1_ (10.41, 95% CI: 6.693∼14.127%, *p* < 0.01) and FVC (9.366, 95% CI: 5.602∼13.131%, *p* < 0.01). In model-2, an increase in 1 unit of the DSP was significantly and negatively associated with the mMRC (−0.769, 95% CI: −0.946∼−0.592, *p* < 0.01), CAT (−3.731, 95% CI: −5.128∼−2.335, *p* < 0.01), and AECOPD (−0.725 times/year, 95% CI: −1.028∼−0.421, *p* < 0.01). As opposed, an increase of 1 unit in the DSP was significantly and positively associated with the FEV1 (10.102, 95% CI: 6.161∼14.044%, *p* < 0.01) and FVC (9.171, 95% CI: 5.325∼13.016%, *p* < 0.01). Regarding linear associations in the LAA%, increases of 1 unit in the LAA% in the total, right, and left lungs were significantly and positively associated with AECOPD (all *p* < 0.05) and FEV_1_ (all *p* < 0.01) n the both mode-1 and model-2.

## 4. Discussion

Patients with COPD typically demonstrate low DSPs, high LAA%, intense dyspnea sensations, declines in their QOL, and increased risks of AECOPD. However, associations among these clinical manifestations remain unclear. This study, hence, employed various grouping approaches to explore the ORs, including between low- and high-DSP groups, between patients with or without severe emphysema (LAA ≥ 20%), and between patients with or without AECOPD. In addition, relationships between pulmonary function measurements and LAA% at various lung volume scales were also investigated.

The low-DSP group exhibited significantly higher values for the mMRC, CAT, AECOPD frequency, and LAA in the lungs (total, right, and left lung regions) compared to those of the high-DSP group. In contrast, patients with low DSP presented significantly lower values of the PFT compared with those with high DSP. This study further investigated the associations (ORs) of all related measurements between the low- and high-DSP groups. The risk of having low DSP was negatively associated with the PFT (OR < 1), whereas it was positively associated with dyspnea sensations, QOL, and AECOPD frequency (ORs > 1). These outcomes may be partially interpreted as individuals with defective lung functional capacity generally demonstrating severe desaturation and poor performance during the 6MWT (22). In other words, poor pulmonary function may have impacted their ability to carry out pacing during the 6MWT due to severe air trapping, dyspnea, and oxygen desaturation. In addition, these manifestations impacted the aforesaid measurements (i.e., the mMRC, CAT, AECOPD, LAA%, and PFT). For instance, a related study documented that the DSP was associated with PFT parameters, dyspnea severity at rest, and the hospitalization rate among their enrolled patients with lung cystic fibrosis (23). Another relevant study suggested that their established models which considered both the DSP and dyspnea severity presented the best ability to predict the health-related QOL for patients with lung sarcoidosis (24). In addition, it was noted that there were significant associations (and with high ORs) of the mMRC, CAT, and AECOPD frequency with grouping by low and high DSPs. These outcomes may also correspond to the 2017 Global Initiative for Chronic Obstructive Lung Disease (GOLD) guidelines that consider respiratory symptoms (i.e., dyspnea sensation, QOL, and exacerbation or hospitalization rate) when classifying COPD severity groups instead of only focusing on PFT parameters (25, 26). Collectively, the current study observed that the DSP, a common and readily determined index, can possibly serve as an indicator for evaluating COPD severity and stability.

This study determined that the LAA% in the lungs (total, right, and left lung regions) was associated with QOL, the AECOPD frequency, PFT, 6-MWT parameters, and DSP. Also, the ORs between the groups with and without severe emphysema (LAA ≥ 20%) of these above variables illustrated similar findings. The likely explanation for these observations is that the LAA, utilized as an indicator of emphysema severity, may enable an assessment of cardiopulmonary function. More precisely, it is plausible that reduced lung function and decreased life quality can be expected in individuals with a high LAA%–namely severe emphysema. Next, it was noted that LAA values were associated with the mMRC, SpO_2_ post-6MWT, and ΔIC between pre- and post-6MWT. These findings also supported the LAA% being related to air trapping, severe dyspnea sensations, and reduced pulmonary gas exchange, which are typical respiratory symptoms in patients with emphysema (27). A similar prior study documented that the LAA% was significantly correlated with declines in the FEV_1_, FVC, and their ratio (FEV_1_/FVC) (20, 28). Associations between the LAA% and derived parameters of the 6MWT, including the lowest SpO_2_ and the ratio of desaturation level to the distance walked during 6MWT among their recruited COPD patients (with a mean LAA of 12.3%), were also observed in a relevant study (29). Altogether, this study determined that a high LAA% corresponded to the DSP and 6MWT performance in our recruited COPD patients.

Previous research suggested that the LAA%, which indicates the severity of emphysema, was correlated with the capacity of lung diffusion and ventilation (30). Unlike previous observations derived from analyzing results of the carbon monoxide diffusing capacity (DLCO), this study determined associations among the LAA%, decreased oxygen desaturation post-6MWT, and declines in the DSP. Pathologically, COPD has been mainly characterized by elevated carbon dioxide levels, caused by parenchymal destruction and reduced elastic alveoli recoil, resulting in air trapping (31). However, some actions caused by emphysema, such as destruction of alveolar-capillary interfaces and increased lung dead space, may partially affect oxygen desaturation (32). A related study documented that the emphysema level was significantly and negatively correlated with the level of arterial oxygen saturation, which may be caused by ventilation-perfusion mismatch (33). Similarly, a relevant study demonstrated that a per unit increase in the LAA% was associated with 10% oxygen desaturation and 20% recurrent oxygen desaturation during the 6MWT in their 283 enrolled COPD patients (34). Taken together, an increase in the LAA%, an indicator of emphysema, may potentially result in ventilation-perfusion disturbance and inhomogeneity of ventilation distribution and, accordingly, cause elevations of carbon dioxide and oxygenation impairment.

Regarding COPD stability, significant associations (ORs) between the risk of having AECOPD, reduced life quality (CAT), worsened dyspnea sensations (mMRC), and declines in PFT measurements (FEV_1_ and FVC) were found. Additionally, the risk of exhibiting AECOPD was negatively associated with the distance walked in the 6MWT and the DSP (ORs < 1), whereas it was positively associated with the LAA% (OR > 1). Previous findings may be partially congruent with ours. For example, one study determined significantly reduced CAT scores between the first and seventh admission days (35). Another study indicated that the mMRC scale and CAT scores were both associated with pulmonary function among patients who were experiencing AECOPD (36). Similarly, another study suggested that approximately one-third of their enrolled patients, with high symptom scores categorized using both the CAT and mMRC scale, demonstrated two instances of AECOPD in 1 year (37). Next, as aforementioned, the LAA% was suggested to be an indicator of emphysema severity and was also correlated with impaired pulmonary function and collapsed airway dimensions (38). These risk factors regarding pulmonary conditions are all related to AECOPD (39). Concerning the association between the DSP and the risk of AECOPD, as mentioned above, patients who demonstrated low DSP values typically had defective pulmonary function, thereby limiting their performance on the 6MWT. A relevant study documented that individuals who had a low DSP usually exhibited a high risk of AECOPD or even hospitalization (9). Another similar study presented that the DSP was a potential indicator to predict whether a patient had pulmonary sarcoidosis (40). Another study reported that the DSP was associated with 1-year mortality in patients who had idiopathic pulmonary fibrosis (41). Taken together, the DSP, LAA, and COPD clinical manifestations, including worsened dyspnea sensation and reduced QOL, were associated with the risk of AECOPD.

The current study has some limitations that should be considered in future analyses. First, since this study only recruited 111 COPD patients and most were male, the derived observations might not be generalizable to other ethnicities. Next, the present study employed a dataset from a single medical institution in northern Taiwan, which may have caused some bias, such as retrospective and survivor bias. The ventilation and perfusion mismatch may decrease oxygen saturation and affect the 6MWT performance (42). However, this study did not obtain results of the diffusion capacity of carbon monoxide for determining the lung ventilation capability, which should be addressed in future work. In terms of confounding factors, although anthropometric features and individuals’ tobacco use status were considered, other factors, such as genes, passive smoking exposure, environmental pollution, and socioeconomic factors, may also affect clinical symptoms of COPD. Another limitation of the current study was that medication usage was undetermined. Specifically, recruited COPD patients were instructed to regularly utilize bronchodilators to maintain their disease stability and their use was only suspended 1 day before the PFT. Measurements of the 6MWT and CT, hence, may have been affected by long-term bronchodilator usage, but we did not record their bronchodilator usage situation. Also, this study did not acquire muscle power or body composition data. However, these details are also related to 6MWT outcomes, especially for individuals with COPD (43). The inflation of odds ratios may potentially affect the obtained outcomes in this retrospective study (44). The effects of these factors should be addressed in future studies.

## 5. Conclusion

Using a COPD dataset from a northern Taiwanese population, this retrospective study observed that a low DSP was significantly associated with a reduced life quality, lower PFT parameters, an elevated dyspnea sensation level, and an increased frequency of AECOPD. Patients in the low-DSP group had significantly higher values of the LAA% in the lungs (total, right, and left lung regions) than did those in the high-DSP group. We observed significant correlations among the QOL, AE frequency, FEV1, ΔIC, DSP, and LAA at various lung volume scales. Next, the risks of exhibiting AECOPD, DSP, and LAA% in various lung regions were determined. Those outcomes confirmed associations of the DSP and LAA% with COPD clinical manifestations (i.e., decreased QOL, worsened dyspnea sensations, increased AECOPD frequency, and reduced PFT). In addition, the DSP may serve as a new indicator to evaluate COPD stability.

## Data availability statement

The raw data supporting the conclusions of this article will be made available by the authors, without undue reservation.

## Ethics statement

The studies involving human participants were reviewed and approved by the Joint Institutional Review Board of Taipei Medical University (TMU-JIRB: N201902008). The patients/participants provided their written informed consent to participate in this study. Written informed consent was obtained from the individual(s) for the publication of any potentially identifiable images or data included in this article.

## Author contributions

S-CH agreed to be accountable for all content and aspects of the work, ensuring that questions related to the accuracy, or integrity of any part of the work are appropriately investigated and revolved. K-YC, H-YK, K-YL, P-HF, S-MW, H-CC, T-TC, W-LS, C-HT, W-TL, W-HC, AM, and MS were involved in the conception and the designs of the study. C-YT and S-CH were responsible for drafting the manuscript. All authors approved the final version of this manuscript and agreed to be accountable for all aspects of the work, full access to all the data in the study and take responsibility for the integrity of the data and the accuracy of the data analysis, involved in data acquisition, analysis, and interpretations, and as well as the critical revision of the manuscript for important intellectual content.
