# Supplementary material for: Associations of the distance-saturation product and low-attenuation area percentage in pulmonary computed tomography with acute exacerbation in patients with chronic obstructive pulmonary disease
Source: Front Med (Lausanne). 2023 Jan 4;9:1047420. doi: 10.3389/fmed.2022.1047420 (PMC9846059; doi:10.3389/fmed.2022.1047420)
Supplement: Supplementary file 1 [file Data_Sheet_1.docx]

**Supplementary table 1. Associations of details of the life quality, dyspnea sensation level, acute exacerbation frequency, and 6-min walk test with low-attenuation area percentage (LAA%) of each lung lobe**

| Categorical variables | Beta coefficient (95% confidence interval) | | | | |
| --- | --- | --- | --- | --- | --- |
|  | Right upper lobe LAA | Right middle lobe LAA | Right lower lobe LAA | Left upper lobe LAA | Left lower lobe LAA |
| mMRC (point) | 3.727 (1.633 to 5.821) ** | 1.772 (0.099 to 3.444) * | 0.922 (-0.487 to 2.33) | 2.733 (0.873 to 4.594) ** | 0.839 (-0.968 to 2.645) |
| CAT (point) | 2.186 (0.096 to 4.275) * | 0.193 (-1.45 to 1.835) | 0.572 (-0.79 to 1.934) | 1.853 (0.026 to 3.679) * | 0.844 (-0.893 to 2.582) |
| AECOPD (times/year) | 2.148 (0.089 to 4.207) * | 1.413 (-0.182 to 3.008) | 1.127 (-0.201 to 2.455) | 2.879 (1.132 to 4.625) ** | 2.724 (1.088 to 4.36) ** |
| Pulmonary function test |  |  |  |  |  |
| FEV_1_ (% predicted) | -4.098 (-6.173 to -2.023) * | -1.484 (-3.172 to 0.205) | -2.549 (-3.884 to -1.214) ** | -4.107 (-5.876 to -2.338) ** | -3.012 (-4.734 to -1.289) ** |
| FVC (% predicted) | -2.16 (-4.345 to 0.026) | -0.22 (-1.935 to 1.494) | -1.044 (-2.456 to 0.367) | -2.314 (-4.204 to -0.424) * | -1.36 (-3.162 to 0.443) |
| 6-min walk test |  |  |  |  |  |
| Distance walked (m) | -3.031 (-5.34 to -0.721) * | -0.542 (-2.376 to 1.292) | 0.154 (-1.372 to 1.681) | -2.052 (-4.094 to -0.009) * | 0.536 (-1.412 to 2.484) |
| SpO_2-pre_ (%) | -0.776 (-2.928 to 1.377) | 0.449 (-1.212 to 2.109) | 0.031 (-1.352 to 1.413) | -0.702 (-2.581 to 1.177) | -0.325 (-2.09 to 1.44) |
| SpO_2-post_ (%) | -3.498 (-5.596 to -1.401) ** | -2.159 (-3.805 to -0.513) * | -1.859 (-3.225 to -0.494) ** | -3.2 (-5.022 to -1.378) ** | -2.496 (-4.234 to -0.758) ** |
| Δ IC (L) | -3.219 (-5.287 to -1.151) ** | -1.581 (-3.218 to 0.055) | -2.166 (-3.485 to -0.847) ** | -2.836 (-4.64 to -1.032) ** | -2.914 (-4.59 to -1.239) ** |
| DSP (m%) | -3.548 (-5.859 to -1.236) ** | -0.979 (-2.828 to 0.87) | -0.213 (-1.758 to 1.332) | -2.573 (-4.619 to -0.527) * | -0.039 (-2.013 to 1.935) |
| Abbreviations: mMRC, modified Medical Research Council; CAT, chronic obstructive pulmonary disease (COPD) assessment test; AE, acute exacerbation; FEV_1_, forced expiratory volume in the first second; FVC, forced vital capacity; SpO_2_, peripheral capillary oxygen saturation; IC, inspiratory capacity; DSP, distance-saturation product.  Multivariable linear regression models were adjusted for age, sex, body-mass index, tobacco use status, and Charlson comorbidity index.  * *p*<0.05; ** *p*<0.01. | | | | | |

**Supplementary table 2. Association summary of independent risk factors for having a low distance-saturation product (DSP)**

| Categorical variables | Low DSP (*N*=37) vs. high DSP (*N*=74) | |
| --- | --- | --- |
|  | Adjusted OR (95% CI) | |
|  | Model 1 | Model-2 |
| mMRC (point) | 4.712 (1.907 to 11.646) ** | 13.548 (3.289 o 55.803) ** |
| CAT (point) | 1.031 (0.933 to 1.139) | 1.021 (0.911 to 1.145) |
| AECOPD (time/year) | 1.109 (0.691 to 1.779) | 1.005 (0.602 to 1.675) |
| Pulmonary function test |  |  |
| FEV_1_ (% predicted) | 0.945 (0.907 to 0.985) ** | 0.913 (0.859 to 0.969) ** |
| LAA (%) |  |  |
| Total lung | 0.978 (0.901 to 1.064) | 0.952 (0.864 to 1.049) |
| Abbreviations: mMRC, modified Medical Research Council; CAT, chronic obstructive pulmonary disease assessment test; AECOPD, acute exacerbation of chronic obstructive pulmonary disease; FEV_1_, forced expiratory volume in the first second; LAA%, low-attenuation area percentage.  The adjusted ORs of model 1 were determined using multivariable logistic regression models adjusted for age, sex, body-mass index, tobacco use status, lung function (FEV_1_), symptoms (mMRC, CAT, and AECOPD), LAA% in the total lungs, and Charlson comorbidity index.  The adjusted ORs of model 2 were determined using multivariable logistic regression models adjusted for age, sex, body-mass index, tobacco use status, lung function (FEV_1_), symptoms (mMRC, CAT, and AECOPD), LAA% in the total lung, and comorbidities (acute myocardial infarction, chronic heart failure, cerebrovascular accident, peripheral vascular disease, hypertension, depression and anxiety, osteoporosis, and metabolic syndrome).  ** *p*<0.01. | | |

**Supplementary table 3. Linear associations of between the alternations in the distance-saturation product (DSP), the details of the life quality, dyspnea sensation level, acute exacerbation frequency, and low-attenuation area percentage (LAA%)**

| Categorical variables | Adjusted Beta coefficient (95% confidence interval) | |
| --- | --- | --- |
|  | Model 1 | Model 2 |
| mMRC (point) | -0.738 (-0.902 to -0.573) ** | -0.769 (-0.946 to -0.592) ** |
| CAT (point) | -3.633 (-4.93 to -2.335) ** | -3.731 (-5.128 to -2.335) ** |
| AECOPD (times/year) | -0.694 (-0.977 to -0.411) ** | -0.725 (-1.028 to -0.421) ** |
| Pulmonary function test |  |  |
| FEV_1_ (% predicted) | 10.41 (6.693 to 14.127) ** | 10.102 (6.161 to 14.044) ** |
| FVC (% predicted) | 9.366 (5.602 to 13.131) ** | 9.171 (5.325 to 13.016) ** |
| LAA (%) |  |  |
| Total lung | -1.922 (-3.597 to -0.247) * | -1.707 (-3.473 to 0.059) |
| Right lung | -1.895 (-3.531 to -0.259) * | -1.768 (-3.501 to -0.034) * |
| Left lung | -1.891 (-3.724 to -0.058) * | -1.599 (-3.517 to 0.318) |
| Abbreviations: mMRC, modified Medical Research Council; CAT, chronic obstructive pulmonary disease assessment test; AE, acute exacerbation; FEV_1_, forced expiratory volume in the first second; FVC, forced vital capacity.  Model 1: The multivariable linear regression models were adjusted for age, sex, body-mass index, tobacco use status, and Charlson comorbidity index.  Model 2: The multivariable linear regression models were adjusted for age, sex, body-mass index, tobacco use status, and comorbidities (acute myocardial infarction, chronic heart failure, cerebrovascular accident, peripheral vascular disease, hypertension, depression and anxiety, osteoporosis, and metabolic syndrome).  * p<0.05; ** p<0.01. | | |

**Supplementary table 4. Linear associations of between the alternations in low-attenuation area percentage (LAA%), the details of the life quality, dyspnea sensation level, acute exacerbation frequency, and 6-min walk test.**

| Categorical variables | Beta coefficient (95% confidence interval) | | | | | |
| --- | --- | --- | --- | --- | --- | --- |
|  | Total lung LAA% | | Right lung LAA% | | Left lung LAA% | |
|  | Model 1 | Model 2 | Model 1 | Model 2 | Model 1 | Model 2 |
| mMRC (point) | 0.316 (0.108 to 0.525) ** | 0.316 (0.094 to 0.539) ** | 0.337 (0.126 to 0.547) ** | 0.325 (0.101 to 0.549) ** | 0.272 (0.067 to 0.477) ** | 0.288 (0.069 to 0.507) * |
| CAT (point) | 1.419 (-0.024 to 2.862) | 1.282 (-0.256 to 2.819) | 1.317 (-0.153 to 2.787) | 1.121 (-0.436 to 2.678) | 1.417 (0.013 to 2.82) * | 1.364 (-0.142 to 2.869) |
| AECOPD (times/year) | 0.427 (0.126 to 0.727) ** | 0.436 (0.117 to 0.755) ** | 0.364 (0.056 to 0.673) * | 0.355 (0.029 to 0.681) * | 0.525 (0.239 to 0.811) ** | 0.563 (0.259 to 0.867) ** |
| Pulmonary function test |  |  |  |  |  |  |
| FEV_1_ (% predicted) | -8.852 (-12.694 to -5.01) ** | -9.213 (-13.159 to -5.268) ** | -8.146 (-12.116 to -4.175) ** | -8.339 (-12.406 to -4.272) ** | -9.037 (-12.739 to -5.335) ** | -9.603 (-13.42 to -5.786) ** |
| FVC (% predicted) | -4.207 (-8.285 to -0.129) * | -5.236 (-9.295 to -1.177) * | -3.644 (-7.807 to 0.52) | -4.474 (-8.609 to -0.339) * | -4.624 (-8.571 to -0.676) * | -5.805 (-9.751 to -1.859) ** |
| 6-min walk test |  |  |  |  |  |  |
| Distance walked (m) | -19.815 (-42.106 to 2.475) | -16.073 (-38.925 to 6.779) | -20.628 (-43.246 to 1.991) | -17.614 (-40.632 to 5.403) | -16.627 (-38.408 to 5.153) | -12.75 (-35.254 to 9.755) |
| SpO_2-pre_ (%) | -0.094 (-0.616 to 0.429) | -0.105 (-0.66 to 0.45) | -0.06 (-0.591 to 0.471) | -0.066 (-0.626 to 0.494) | -0.169 (-0.677 to 0.339) | -0.19 (-0.734 to 0.353) |
| SpO_2-post_ (%) | -1.875 (-2.961 to -0.788) ** | -1.859 (-3.019 to -0.699) ** | -1.85 (-2.957 to -0.743) ** | -1.826 (-3.0 to -0.653) ** | -1.867 (-2.922 to -0.813) ** | -1.866 (-3.001 to -0.73) ** |
| ΔIC (L) | -0.1 (-0.154 to -0.047) ** | -0.101 (-0.157 to -0.044) ** | -0.094 (-0.149 to -0.039) ** | -0.095 (-0.152 to -0.037) ** | -0.097 (-0.149 to -0.044) ** | -0.097 (-0.153 to -0.041) ** |
| DSP (m%) | -23.889 (-44.704 to -3.075) * | -20.722 (-42.163 to 0.719) | -24.469 (-45.597 to -3.342) * | -22.018 (-43.608 to -0.427) * | -20.993 (-41.347 to -0.64) * | -17.634 (-38.775 to 3.506) |
| Abbreviations: mMRC, modified Medical Research Council; CAT, chronic obstructive pulmonary disease assessment test; AE, acute exacerbation; FEV_1_, forced expiratory volume in the first second; FVC, forced vital capacity; SpO_2_, peripheral capillary oxygen saturation; IC, inspiratory capacity; DSP, distance-saturation product.  Model 1: The multivariable linear regression models were adjusted for age, sex, body-mass index, tobacco use status, and Charlson comorbidity index.  Model 2: The multivariable linear regression models were adjusted for age, sex, body-mass index, tobacco use status, and comorbidities (acute myocardial infarction, chronic heart failure, cerebrovascular accident, peripheral vascular disease, hypertension, depression and anxiety, osteoporosis, and metabolic syndrome).  * p<0.05; ** p<0.01. | | | | | | |
